# Use of Phentolamine Mesylate in Implant Surgery: Analysis of Adverse Effects and Haemodynamic Changes

**DOI:** 10.3390/jcm10173875

**Published:** 2021-08-28

**Authors:** Clara Vintanel-Moreno, José María Martínez-González, Natalia Martínez-Rodríguez, Cristina Meniz-García, Isabel Leco-Berrocal

**Affiliations:** Department of Dental Clinical Specialties, Faculty of Dentistry, Complutense University of Madrid, 28040 Madrid, Spain; clara.vintanel@gmail.com (C.V.-M.); jmargo@ucm.es (J.M.M.-G.); hospinatmr@hotmail.com (N.M.-R.); mileco@ucm.es (I.L.-B.)

**Keywords:** phentolamine mesylate, dental implants, haemodynamic changes, adverse effects, satisfaction

## Abstract

The clinical application of phentolamine mesylate (PM) as an anaesthetic reversal agent has been documented in the paediatric population and in conservative dentistry, but no studies have been conducted regarding dental implant surgery. A prospective randomised study was conducted on 60 patients eligible for mandibular implant treatment, randomly divided between a control group (CG) and an experimental group (EG), to whom PM was administered. Haemodynamic changes, adverse effects and patient satisfaction were assessed. No statistically significant differences in haemodynamic changes and postoperative pain were found between CG and EG (*p* < 0.05), except for systolic blood pressure (SBP), which increased slightly in EG, without posing a risk to patients. There were no differences in the occurrence of adverse effects between the two groups, except for greater difficulty in chewing and biting (*p* < 0.05) in CG and greater pain in the injection area (*p* = 0.043) in EG. Among EG patients, 83.3% reported that they would request PM again for future dental treatment. The use of PM offers an alternative to implant surgery, thereby increasing patients’ quality of life without increasing the risks.

## 1. Introduction

Local anaesthetics used in the oral cavity often last much longer than the working time, which can lead to decreased quality of life of patients who undergo dental treatment.

All anaesthetic techniques used produce total or partial loss of soft tissue sensitivity for 3 to 5 h [1,2,3,4], which is often longer than the working time plus the time needed for pain control after a restorative or periodontal procedure [5,6]. This duration is most often associated with difficulty in eating, drinking, speaking and smiling [7,8,9,10,11], and can cause biting injuries to the lips, tongue and cheeks, particularly in children and disabled people [12,13]. Some patients consider it to be a temporary decrease in their quality of life [7,12,14], affecting their normal daily activity in three areas: perceptual (altered perception of physical appearance), sensory (lack of sensation) and functional (impaired ability to speak, smile, drink and control salivation) [5,14,15,16].

The use of an anaesthetic reversal agent, such as PM, has been well documented in the literature. In dentistry, such agents are used to eliminate the anaesthetic in the area where it has been injected. This is achieved via vasodilatation, owing to the increase in blood flow [17]. PM is not an anaesthetic antagonist, but an antagonist of the vasoconstrictor with which the anaesthetic is associated. Therefore, it should not be used to reverse anaesthetics that are not associated with a vasoconstrictor [18].

PM is a non-specific α-adrenergic inhibitor whose effect is vasodilation of the vascular system [7,8,11,17]. It inhibits the ability of sympathomimetic amines, such as norepinephrine and epinephrine, to stimulate vascular contraction [19,20]. Given that it is an α-adrenergic inhibitor, special attention should be paid to any cardiovascular changes that may occur, such as hypotension and/or tachycardia following the injection [9]. It is believed that increased blood flow occurs due to vasodilatation in the injection site, which then results in the anaesthetic being eliminated from the area [17].

Tavares et al. [21] reported on the clinical application of PM in paediatric patients. The study shows a decrease in the duration of the anaesthetic effect after restorative and hygienic treatment. The main adverse effects found are pain at the site of injection, minor alterations in vital signs and post-treatment pain. Nourbakhsh et al. [5] also analysed the effect of PM in routine dental treatments in children aged 4 to 11 years anaesthetised with lidocaine 2% with epinephrine 1:80,000. The study found statistically significant differences in the duration of the anaesthetic effect between the PM group and the placebo group. The adverse effects included one incident of nausea and one of a rise in temperature, both after PM injection.

In adult patients, Fowler et al. [15] analysed the usefulness of PM in maxillary or mandibular endodontic treatment of asymptomatic teeth, using lidocaine 2% with epinephrine 1:100,000 in all cases. Differences in the duration of the anaesthetic effect were confirmed. The main adverse effect was subjective intraoral swelling in the PM group.

There are many studies using PM in different dental procedures, but not in implantology, thus this study was developed to address the current demand for treatment within the field. The objectives were to analyse the haemodynamic changes and the occurrence of adverse effects attributable to PM, and assess the recommendation for its use in adult patients undergoing implant treatment.

## 2. Materials and Methods

### 2.1. Study Design and Patient Selection

A prospective longitudinal study was designed at the Faculty of Dentistry at Complutense University of Madrid, with approval by the Ethics Committee of the Hospital Clínico San Carlos (18/011-O_EPASP) and following the recommendations of the Declaration of Helsinki.

The study included patients who requested implant treatment and required the placement of two mandibular implants in premolar and molar positions. The inclusion criteria were as follows: patients over 18 years of age; belonging to the ASA 1 group; voluntary participation in the study; anaesthetic latency time of less than 4 min (measured from the start of the injection of anaesthetic solution until the patient felt lip numbness); and diagnostic CBCT prior to implant placement (iCAT Next Generation, Imaging Sciences International, Inc., Hatfield, PA, USA).

This three-dimensional study assessed whether bone availability was sufficient to place a dental implant, which maintained 1.5–2 mm of bone in the vestibular area and –1.5 mm in the lingual area. If this was not the case, those patients were excluded from the study, as bone regeneration was required either prior to or simultaneous with implant placement. Patients whose questionnaire was incomplete; patients with ASA class II, III or IV; patients for whom more than 2 carpules of anaesthetic were necessary for treatment; patients with arterial hypertension (AHT) or alterations in heart rate (HR); patients who chronically take anti-inflammatory and/or analgesic drugs; patients who take any other type of medication; and those who did not wish to participate in the study were excluded.

Inclusion was by probability sampling of consecutive cases, counting all patients who met the inclusion criteria and voluntarily agreed to participate in the study. To ensure consent, patients were given an information sheet, and the purpose of the procedure, including possible adverse effects, was explained verbally. They then signed an informed consent form prior to the procedure.

A total of 60 patients were included, who were randomly distributed to the control group (CG) and experimental group (EG) (30 in each). A single clinician (C.V.M.) performed all surgeries. Randomisation was performed by another researcher (I.L.B.), using identical opaque envelopes whose content determined group assignation as CG or EG. Neither the clinician nor the patients were aware of which group they had been randomised to until the sutures were applied. After each surgery was concluded, the envelope was opened and the clinician and patient were informed of the group assignment.

All patients were fitted with two BioHorizons^®^ brand dental implants (Tapered Internal with Laser-Lok, BioHorizons Birmingham, AL, USA) of a suitable diameter and length according to the bone availability shown on CBCT.

### 2.2. Surgical Technique

Implant placement was always performed by the same surgeon and following the same surgical protocol. An inferior alveolar nerve (IAN) and lingual nerve block was performed, followed by buccal nerve reinforcement using a 1.7 mL carpule of 4% articaine with adrenaline 1:100,000 (Septanest^®^ 1/100,000, Septodont, France) in each injection and rinsing with chlorhexidine digluconate 0.20% (Lacer^®^ chlorhexidine; Barcelona, Spain) for 60 s.

A crestal incision was made and a full-thickness mucoperiosteal flap was raised, drilling was performed with abundant flushing to avoid bone necrosis, and the implants were mechanically placed. The incision area was sutured with 4/0 silk using simple stitches (Supramid^®^ Aragó^®^, Barcelona, Spain). Once the procedure was finished, a 1.7 mL carpule of PM (OraVerse^®^; Septodont, Saint-Maur-des-Fosses, France) was injected near the IAN block injection zone.

A data collection form was used to record the start time of the procedure prior to the injection of anaesthetic solution, the time when the patient began to feel lip numbness (latency period, from the start of injection until the patient felt numbness), and the end of the procedure, measured after suturing was completed.

### 2.3. Data Recording and Monitoring

In order to cover the primary objectives of this study, all patients were monitored by measuring their systolic blood pressure (SBP) and diastolic blood pressure (DBP) using a blood pressure monitor (Omron M3 Comfort^®^ HEM-7155-E; Omron Healthcare Europe, Hoofddorp, The Netherlands), and HR and O_2_ saturation (SaO_2_) using a handheld Bippex pro Apex Medical^®^ digital pulse oximeter (Iberomed, Spain). In both groups, recordings were taken prior to the injection of anaesthetic solution to obtain a baseline measurement, and 10 min after the end of the procedure in CG and 10 min after PM injection in EG. Patients were monitored at 24, 48 and 96 h after the injection for adverse effects and after 7 days for suture removal.

Patients were given a sheet with instructions to follow in the days following the procedure, and all were prescribed amoxicillin 750 mg, 1 every 8 h for 7 days, and paracetamol 650 mg as analgesic treatment, if necessary. They were requested to complete an 11-item questionnaire, which included clinical aspects on the consequences of the anaesthetic effects and possible adverse effects, and return it 24 h after the procedure. This questionnaire assessed, among other aspects, whether patients had experienced speech disorders, unusual sensations, palpitations or dizziness, any neck or head pain, etc. The responses were recorded using a Likert-type scale, with values of 0 (no), 1 (minimally), 2 (sometimes), 3 (quite a lot) and 4 (always).

Regarding the secondary outcomes, all patients were instructed to write down the time when lip or tongue numbness disappeared. Patients were also given a questionnaire in which they were asked to record the intensity of pain using a visual analogue scale (VAS) (0–10) and the number of analgesics taken 6, 12 and 24 h after the end of the procedure, as well as in the following days.

After 7 days and coinciding with suture removal, participants were given a new 3-item questionnaire, taken from Saunders et al. [6], in which they were asked the following: (1) If you were to have the same procedure, now that you are aware of the existence of this product, would you request it? (2) Did you feel an increase in your overall satisfaction after the procedure due to the reduction in the anaesthetic effect time? (3) Would you recommend the use of this product to family and friends? The answers for each question were no, yes, don’t know or no response.

### 2.4. Statistical Analysis

The data collected were transcribed into an Excel spreadsheet (MS Excel 2007, Microsoft Inc., Redmond, WA, USA). An independent statistician analysed the data with specialised software (SPSS Statistics, version 25.0, IBM Corp., Armonk, NY, USA). In the first phase, a descriptive study of frequencies was carried out, in which the mean, median, standard deviation and ranges were obtained. To compare the groups with quantitative variables, normality was first checked with the Shapiro–Wilk test statistic. Student’s t-test or the non-parametric Mann–Whitney U-test was performed when normality was rejected. For intra-class comparison of quantitative variables at 2 moments (pre- and post-surgery), the non-parametric Wilcoxon test was used. The study of qualitative variables was carried out using Pearson’s chi-square test. A significance level of *p* ≤ 0.05 was set.

## 3. Results

A total of 60 patients were included in the present study, 30 in CG (control) and 30 in EG (OraVerse^®^). The gender distribution of the participants was 22 females (36.7%) and 38 males (63.3%), with an F/M ratio of 1/1.7. The mean age of patients was 54.78 ± 11.34 years.

The mean duration of the procedure in both groups was 25.13 ± 7.04 min, with a duration of 25.58 ± 7.63 min in CG and 24.68 ± 6.49 min in EG. There were no significant differences in latency or procedure time between the two groups.

In CG at baseline, mean SBP was 128.37 ± 14.225, mean DBP was 78.50 ± 7.826, HR was 74.93 ± 11.867 and SaO_2_ was 99%. In EG, SBP was 121.40 ± 11.325, DBP was 78.27 ± 7.395, HR was 75.73 ± 8.944 and SaO_2_ was 98.99 ± 0.254%. Ten minutes after the end of the procedure, the mean results obtained after measurement in CG were SBP 128.40 ± 14.98, DBP 80.87 ± 10.05, HR 72.33 ± 10.16 and SaO_2_ 99.00%, and in CG were SBP 125.20 ± 11.59, DBP 79.47 ± 8.11, HR 74.47 ± 8.67 and SaO_2_ 98.99 ± 0.25%. Comparing the two groups, no significant differences were found (Table 1).

Baseline values between the two groups only differed in SBP, which was higher in CG (*p =* 0.041). Comparing baseline versus final results, significant differences were once again found in SBP, with an increase at the final recording compared to baseline in EG (*p* = 0.011). There was a decrease in HR at the final recording compared to baseline in CG (*p* = 0.020).

**Figure 1:**
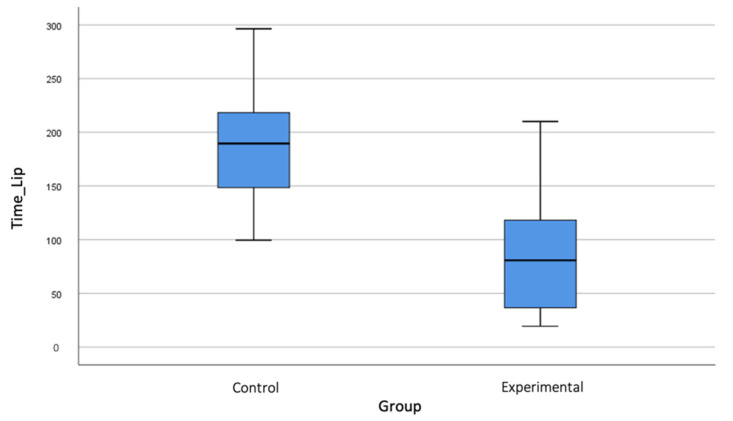
Bloxplot showing differences between control and experimental groups in lip numbness recovery time.

**Figure 2:**
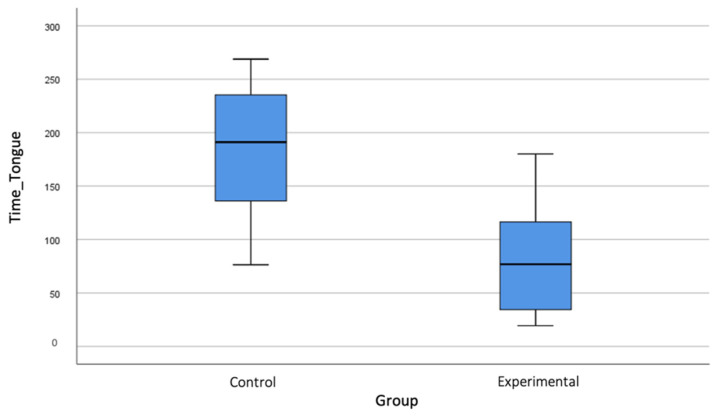
Bloxplot showing differences between control and experimental groups in tongue numbness recovery time.

The duration of the anaesthetic effect at the labial level (Figure 1) was longer in CG, with a mean of 190.05 ± 49.99 min, compared to 87.14 ± 53.36 min for EG (*p* < 0.001). This difference was also observed in the recovery of tongue sensation (Figure 2), with values of 180.21 ± 61.63 min for CG versus 78.27 ± 44.92 min for EG (*p* < 0.001).

**Figure 3:**
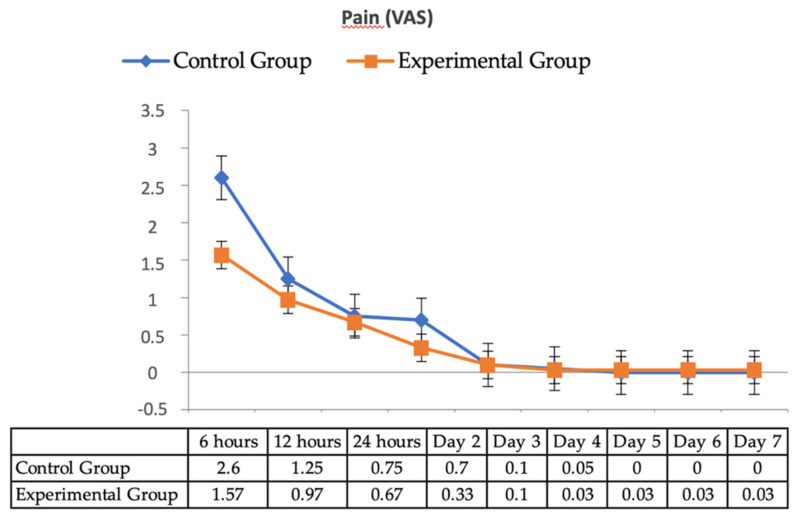
Mean values of pain intensity recorded by VAS.

**Figure 4:**
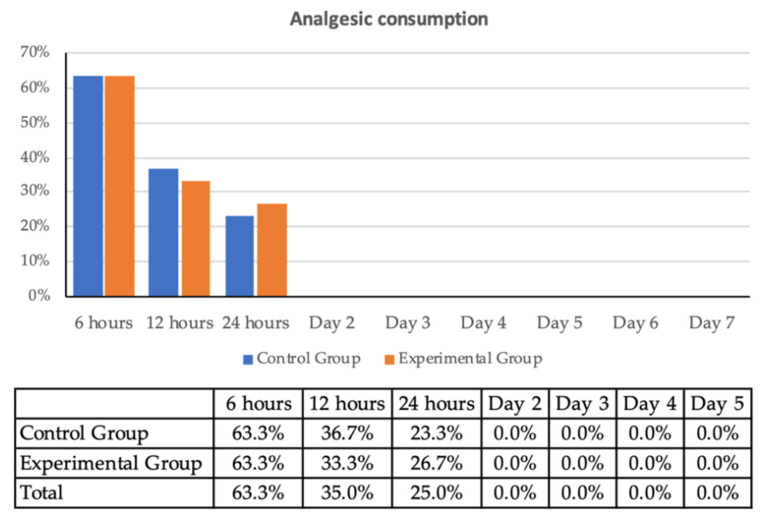
Percentage of patients who used analgesics.

Postoperative pain intensity recorded by VAS (Figure 3) showed that in both groups, the pain response was similar and of moderate intensity, with values between 0 and 5. A similar trend was observed with the use of analgesics during the first 24 h and the absence of pain in the control group (Figure 4).

An analysis of the postoperative clinical questionnaire collected at 24 h (Table 2) showed statistically significant differences in relation to difficulty in chewing (*p* = 0.01) and accidental bites (*p* = 0.05), which were greater in CG. On the contrary, greater pain in the anaesthetic area was recorded in EG (*p* = 0.043).

Regarding possible adverse effects of OraVerse^®^, no statistically significant values were observed between the two groups. Speech impairment (*p* = 0.633), nausea (*p* = 0.402), feeling of palpitation or dizziness (*p* = 0.31) and head or neck pain (*p* = 0.393) did not differ between the two groups.

In the three-item questionnaire asking about satisfaction with OraVerse^®^, 83.3% of patients reported that they would request it again for other dental procedures, 76.1% were satisfied with the reduction in numbness time and 83.3% would recommend it to family and friends.

## 4. Discussion

PM quickly reverses the anaesthetic effect, thus avoiding lip and cheek bites, speech impairment and, ultimately, reduced quality of life for patients [11,12,22]. Studies thus far have been mainly conducted on its use after conservative dentistry treatment, but not after surgery or dental implants.

According to Hersh et al. [9], the occurrence of haemodynamic adverse effects due to the use of PM can affect up to one in 10 patients, with a decrease or increase in heart rate and an increase in blood pressure.

Laviola et al. [10] observed in their study that the most frequent adverse effect, in both patients injected with PM and a control group injected with placebo in similar proportions, was tachycardia, which in most cases occurred 10 min after the injection. Therefore, they concluded that one or two injections of 0.4 mg PM would be well tolerated, given the similar trend for patients treated with PM or placebo. In contrast, in the present study, a slight decrease in heart rate was observed between the baseline and final measurements across both groups, although these results were not significant.

With regard to blood pressure, differences were only observed in SBP, which was increased 10 min after PM injection in EG. Despite this difference, the mean SBP was 125.20 mm Hg, which was lower than the mean SBP of 128.40 mm Hg measured 10 min after the end of the procedure in CG, and does not represent a risk for patients. These results are in line with those reported by Daubländer et al. [23], who, following a multicentre study, concluded that the use of PM in routine dental treatment was well tolerated, effective and safe. However, these results contrast those obtained by Hersh et al. [9], who reported an increase of 20 mm Hg in both SBP and DBP in EG, and a decrease in HR by 20 beats/minute. The difference in results could be attributed to the latter study being conducted on a paediatric population.

In the literature reviewed, adverse effects such as pain in the area of the PM injection, trismus, and biting of the lip, tongue or cheek are also reported for some patients [24,25]. In a systematic review and meta-analysis, Vinnakota et al. [26] noted that although no serious complications were reported with the use of PM, mild or moderate adverse effects were reported compared to the control group, with age considered a significant factor. The results of the patient questionnaire in the present study, which assessed the presence of adverse effects, show no differences between EG and CG with regard to the presence of effects such as speech difficulty, headache or neck pain, nausea, vomiting or palpitations.

Most studies on the use of PM in dentistry are related to conservative dentistry and non-invasive periodontal treatment. In a study carried out by Froum et al. [27] on 10 patients undergoing dental implant surgery, anaesthetic effect reversal was used to assess early lower alveolar nerve involvement by measuring the time to recovery of lip and tongue sensation, the presence of bleeding at the site of surgery, and redness and swelling at the site of injection of the anaesthetic solution and PM. No bleeding was observed in any of the 10 patients, and six of them had redness and swelling at the injection site. Michaud et al. [17] found that more patients in the experimental group experienced pain at the injection site, but there were no statistically significant differences with the control group. Their results are similar to those reported in this study; although no side effects of interest were found, patients in the EG did present greater pain in the injection area. This can be explained by the reversal of the anaesthetic effect due to the blocking of adrenergic activity and the additional volume of solution administered [28].

Regarding the duration of the anaesthetic effect on the tongue and lip, the results of this study show a significant reduction in the loss of anaesthetic sensation, agreeing with the results reported in some studies [7,14,16]. The main difference lies in the recovery times, which were somewhat shorter in studies such as [7], which reported an average of 70 min for lip and 60 min for tongue recovery. Fowler et al. [15] observed a lip sensation recovery time of 47 min, although this shorter time could be explained by their use of lidocaine; in contrast, the present study used articaine, which lasts longer.

In a recent randomised double-blind clinical trial using three local anaesthetics (lidocaine, articaine and bupivacaine) associated with a vasoconstrictor published by Gago et al. [29], it was found that lip and tongue sensation recovery time was slightly higher than that observed in this present study: 88.5 min for the lip and 84.5 min for the tongue. As there was no control group, their results were extrapolated from the duration times described by the manufacturers and reported in the literature. In a new randomised controlled clinical trial conducted with bupivacaine, Michaud et al. [30] also concluded that the use of PM significantly reduced the duration of the anaesthetic effect.

As for the presence of postoperative pain, which is a common symptom of surgical procedures in the oral cavity, no significant differences were found between EG and CG. Therefore, although most studies with PM are conducted with conservative and paediatric treatments, PM could be used in surgical and implant treatments, as the reversal of the anaesthetic effect did not lead to an increase in postoperative pain or analgesic consumption as compared to controls.

Regarding patient satisfaction with the use of PM, a study by Saunders et al. [6] showed a positive assessment, with 79% of patients reporting that they would opt for the use of PM in future treatments, 84% considering it a better dental experience and 83% saying they would recommend it to family and friends. These authors also reported dentist satisfaction, which was 86 and 82%, respectively, and stated that PM met a patient need in their clinics and fulfilled its expectations. These results are similar to those of the present study, as patients also reported a high degree of satisfaction with the use of PM, although somewhat less compared to the results of Gago et al. [29], with 98.9% of patients reporting that they would recommend PM.

## 5. Conclusions

The use of PM in the placement of dental implants does not pose a risk of adverse cardiovascular effects in patients or increase postoperative pain, but it does improve patient satisfaction. However, randomised studies with larger numbers of participants and systemic problems would be needed to confirm the safety of PM.

## Figures and Tables

**Table 1:** Differences in mean and statistical significance (*p* < 0.05) in both groups between baseline and final values.

Baseline Values								
	SBP	*p*	DBP	*p*	HR	*p*	SaO_2_	*p*
CG	128.37	0.041 *	78.50	0.905	74.93	0.754	99%	0.155
EG	121.40		78.27		75.73		98.99%	
**Final Values**								
	SBP	*p*	DBP	*p*	HR	*p*	SaO_2_	*p*
CG	128.40	0.341	80.87	0.550	72.33	0.373	99%	0.155
EG	125.20		79.47		74.47		98.99%	

CG, control group; EG, experimental group; SBP, systolic blood pressure; DBP, diastolic blood pressure; HR, heart rate; SaO_2_, O_2_ saturation. * *p* < 0.05.

**Table 2:** Clinical aspects and adverse effects collected during first 24 h postoperatively.

	Questions		0	1	2	3	4	Average	*p*
1	Have you experienced speech impairment?	CG	60%	30%	5%	5%		0.55	0.253
		EG	76.7%	13.3%	10%			0.33	
2	Have you had any difficulty eating?	CG	20%	60%	10%	10%		1.10	0.01 *
		EG	73.3%	13.3%	13.3%			0.40	
3	Have you bitten your tongue, lip or cheek during this time?	CG	80%	5%	15%			0.35	0.05 *
		EG	96.7%	3.3%				0.03	
4	Have you experienced more salivation than usual?	CG	75%	10%	10%	5%		0.45	0.703
		EG	66.7%	26.7%	3.3%	3.3%		0.43	
5	Have you felt unwell? (headache, nausea, etc.)	CG	95%		5%			0.10	0.361
		EG	86.7%	10%			3.3%	0.23	
6	Have you had difficulty communicating?	CG	80%	20%				0.20	0.523
		EG	73.3%	20%	6.7%			0.33	
7	Have you felt a temporary decrease in your quality of life?	CG	55%	40%		5%		0.60	0.084
		EG	80%	13.3%	3.3%	3.3%		0.30	
8	Have you had pain in the area where the anaesthetic solution was injected?	CG	70%	30%				0.30	0.043 *
		EG	46.7%	30%	16.7%	3.3%	3.3%	0.87	
9	Have you noticed palpitations or a dizzy feeling? (heart rate)	CG	100%					0.00	0.414
		EG	96.7%	3.3%				0.03	
10	Have you had a headache or neck pain? (blood pressure)	CG	90%	10%				0.10	0.725
		EG	86.7%	13.3%				0.13	
11	Have you had an itching, tingling or burning sensation in the area around the injection site?	CG	75%	25%				0.25	0.191
		EG	60%	26%	10%	3.3%		0.57	

CG, control group; EG, experimental group. * *p* < 0.05.

## Data Availability

The databases used and/or analysed during the current study are available from the corresponding author upon reasonable request.

## References

[1] Yang F., Gao Y., Zhang L., Zheng B., Wang L., Sun H., Huang D. (2020). Local anaesthesia for surgical extraction of mandibular third molars: A systematic review and network meta-analysis. Clin. Oral Investig..

[2] Camps-Font O., Figueiredo R., Sánchez-Torres A., Clé-Ovejero A., Coulthard P., Gay-Escoda C., Valmaseda-Castellón E. (2020). Which is the most suitable local anaesthetic when inferior nerve blocks are used for impacted mandibular third molar extraction? A network meta-analysis. Int. J. Oral Maxillofac. Surg..

[3] Al-Shayyab M.H., Baqain Z.H. (2018). Factors predictive of the onset and duration of action of local anesthesia in mandibular third-molar surgery: A prospective study. Eur. J. Oral Sci..

[4] Boonsiriseth K., Chaimanakarn S., Chewpreecha P., Nonpassopon N., Khanijou M., Ping B., Wongsirichat N. (2017). 4% lidocaine versus 4% articaine for inferior alveolar nerve block in impacted lower third molar surgery. J. Dent. Anesth. Pain Med..

[5] Nourbakhsh N., Shirani F., Babaei M. (2012). Effect of phentolamine mesylate on duration of soft tissue local anesthesia in children. J. Res. Pharm. Pract..

[6] Saunders T.R., Psaltis G., Weston J.F., Yanase R.R., Rogy S.S., Ghalie R.G. (2011). In-practice evaluation of OraVerse for the reversal of soft-tissue anesthesia after dental procedures. Compend. Contin. Educ. Dent..

[7] Hersh E.V., Moore P.A., Papas A.S., Goodson J.M., Navalta L.A., Rogy S., Rutherford B., Yagiela J.A. (2008). Reversal of soft-tissue local anesthesia with phentolamine mesylate in adolescents and adults. J. Am. Dent. Assoc..

[8] Rutherford B., Zeller J.R., Thake D. (2009). Local and systemic toxicity of intraoral submucosal injections of phentolamine mesylate (OraVerse). Anesth. Prog..

[9] Hersh E.V., Lindemeyer R., Berg J.H., Casamassimo P.S., Chin J., Marberger A., Lin B.P., Hutcheson M.C., Moore P.A. (2017). Phase four, randomized, double-blinded, controlled trial of phentolamine mesylate in two- to five-year-old dental patients. Pediatr. Dent..

[10] Laviola M., McGavin S.K., Freer G.A., Plancich G., Woodbury S.C., Marinkovich S., Morrison R., Reader A., Rutherford R.B., Yagiela J.A. (2008). Randomized study of phentolamine mesylate for reversal of local anesthesia. J. Dent. Res..

[11] Morrow T. (2008). OraVerse helps you lose that numbing feeling. Manag. Care.

[12] Moore P.A., Hersh E.V., Papas A.S., Goodson J.M., Yagiela J.A., Rutherford B., Rogy S., Navalta L. (2008). Pharmacokinetics of lidocaine with epinephrine following local anesthesia reversal with phentolamine mesylate. Anesth. Prog..

[13] Haghighat A., Davoudi A., Minaiyan M., Molai M., Afshar A., Basiri K. (2015). Effect of a trial pharmaceutical solution on reversing sensations after using lidocain: An animal study. Anesth. Essays Res..

[14] Prados-Frutos J.C., Rojo R., Gonzalez-Serrano J., Gonzalez-Serrano C., Sammartino G., Martinez-Gonzalez J.M., Sánchez-Moncescillo A. (2015). Phentolamine mesylate to reverse oral soft-tissue local anesthesia: A systematic review and meta-analysis. J. Am. Dent. Assoc..

[15] Fowler S., Nusstein J., Drum M., Reader A., Beck M. (2011). Reversal of soft-tissue anesthesia in asymptomatic endodontic patients: A preliminary, prospective, randomized, single-blind study. J. Endod..

[16] Elmore S., Nusstein J., Drum M., Reader A., Beck M., Fowler S. (2013). Reversal of pulpal and soft tissue anesthesia by using phentolamine: A prospective randomized, single-blind study. J. Endod..

[17] Michaud P.L., Flood B., Brillant M.S. (2018). Reversing the effects of 2% lidocaine: A randomized controlled clinical trial. J. Dent..

[18] Wynn R.L. (2009). Phentolamine mesylate—An old medical drug becomes a new dental drug. Gen. Dent..

[19] Grover H.S., Gupta A., Saksena N., Saini N. (2015). Phentolamine mesylate: It’s role as a reversal agent for unwarranted prolonged local analgesia. J. Indian Soc. Pedod. Prev. Dent..

[20] Yagiela J.A. (2011). What’s new with phentolamine mesylate: A reversal agent for local anaesthesia?. SAAD Dig..

[21] Tavares M., Goodson J.M., Studen-Pavlovich D., Yagiela J.A., Navalta L.A., Rogy S., Rutherford B., Gordon S., Papas A.S. (2008). Reversal of soft-tissue local anesthesia with phentolamine mesylate in pediatric patients. J. Am. Dent. Assoc..

[22] Helmi M., AlDosari M., Tavares M. (2018). Phentolamine Mesylate may be a safe and effective option to reduce discomfort and time to recovery after dental care with local anesthesia. J. Evid. Based. Dent. Pract..

[23] Daubländer M., Liebaug F., Niedeggen G., Theobald K., Kürzinger M.L. (2017). Effectiveness and safety of phentolamine mesylate in routine dental care. J. Am. Dent. Assoc..

[24] Boynes S.G., Riley A.E., Milbee S., Bastin M.R., Price M.E., Ladson A. (2013). Evaluating complications of local anesthesia administration and reversal with phentolamine mesylate in a portable pediatric dental clinic. Gen. Dent..

[25] Prasanna J.S. (2012). OraVerse: Reverses numbness after dental procedures. J. Maxillofac. Oral Surg..

[26] Vinnakota D.N., Kamatham R. (2019). Safety profile of phentolamine mesylate as reversal agent of pulpal and soft tissue dental anesthesia: A systematic review and meta-analysis. Quintessence Int..

[27] Froum S.J., Froum S.H., Malamed S.F. (2010). The use of phentolamine mesylate to evaluate mandibular nerve damage following implant placement. Compend. Contin. Educ. Dent..

[28] Hersh E.V., Lindemeyer R.G. (2010). Phentolamine mesylate for accelerating recovery from lip and tongue anesthesia. Dent. Clin. N. Am..

[29] Gago-García A., Barrilero-Martin C., Alobera-Gracia M.A., Del Canto-Pingarrón M., Seco-Calvo J. (2021). Efficacy of phentolamine mesylate in reducing the duration of various local anesthetics. J. Dent. Anesth. Pain Med..

[30] Michaud P.L., Nowe E., Smith Brillant M. (2020). Reversing the effects of 0.5% bupivacaine using phentolamine mesylate: A preliminary randomized controlled clinical trial. J. Clin. Pharmacol..

